# Molecular Dynamics Model to Explore the Initial Stages of Anion Exchange involving Layered Double Hydroxide Particles

**DOI:** 10.3390/nano12224039

**Published:** 2022-11-17

**Authors:** Gerard Novell Leruth, Alena Kuznetsova, João Tedim, José R. B. Gomes, Tiago L. P. Galvão

**Affiliations:** 1 CICECO-Aveiro Institute of Materials, Department of Chemistry, University of Aveiro, Campus Universitário de Santiago, 3810-193 Aveiro, Portugal; 2 CICECO-Aveiro Institute of Materials, Department of Materials and Ceramic Engineering, University of Aveiro, Campus Universitário de Santiago, 3810-193 Aveiro, Portugal; 3 Smallmatek—Small Materials and Technologies Lda., Rua dos Canhas, 3810-075 Aveiro, Portugal

**Keywords:** layered double hydroxides, molecular dynamics, anion exchange, delamination, corrosion inhibitor, self-healing

## Abstract

A classical molecular dynamics (MD) model of fully unconstrained layered double hydroxide (LDH) particles in aqueous NaCl solution was developed to explore the initial stages of the anion exchange process, a key feature of LDHs for their application in different fields. In particular, this study focuses on the active corrosion protection mechanism, where LDHs are able to entrap aggressive species from the solution while releasing fewer corrosive species or even corrosion inhibitors. With this purpose in mind, it was explored the release kinetics of the delivery of nitrate and 2-mercaptobenzothiazole (MBT, a typical corrosion inhibitor) from layered double hydroxide particles triggered by the presence of aggressive chloride anions in solution. It was shown that the delamination of the cationic layers occurs during the anion exchange process, which is especially evident in the case of MBT^−^.

## 1. Introduction

Layered double hydroxides (LDHs) are clay-based structures that can be represented by the formula [M^II^_1-*x*_ M^III^*_x_* (OH)_2_]*^x+^* (A*^y^*)*_x/y_* · *z*H_2_O, where M^II^ and M^III^ are divalent and trivalent metal cations, respectively, and A*^y^* is a *y*-valence anion [1]. In the case of synthetic LDHs, cations such as Zn^2+^ and Mg^2+^ are most commonly used as M^II^, while Al^3+^ is quite popular as M^III^. The metal hydroxide layers have an overall positive charge (*x*+), which is balanced by interlayer anions solvated by a certain amount of water molecules that stabilize the structure via hydrogen bonding. This results in the capacity to intercalate different types of anionic species (from small inorganic or organic species to large biomolecules, such as DNA [2]). Some important aspects of the structure are still unclear, which can vary with different characteristics associated with the cationic layer and interlayer anions, such as the stacking arrangement of the layers, the position of the anions in the interlayer, the ordering of the metallic atoms in the cationic layer, and the number of water molecules in the interlayer [3]. Computational techniques, such as density functional theory (DFT) [4,5] and classical MD [6,7], have been playing a key role in exploring the inner structure of LDHs.

LDHs find applications in a broad range of fields, such as catalysis [8], adsorption of hazardous species [9,10], drug delivery [11,12], water purification [13], or corrosion sensing [14] and protection [1]. In the case of the latter, LDHs were found to efficiently protect metallic surfaces, either through the formation of conversion films [15] or incorporated as functional additives in organic coatings [16], thus decreasing the permeability of the polymeric coatings to corrosive species, such as chlorides [16]. The corrosion protection is potentiated by the ability of LDHs to act as reservoirs of functional molecules (e.g., corrosion inhibitors) in the anionic form, which are released in a controlled manner upon exchange with aggressive species, also in the anionic form, such as, for example, chlorides. The delivery process has been suggested to occur via one of the following [17]: (i) simple diffusion mechanism [18]; (ii) delamination of the LDH cationic layers [19]; or (iii) reconstruction of the LDH material [20]. Therefore, since the mechanism of anion exchange in LDHs is still not clearly understood, it is reported in this work a classical MD model of fully unconstrained LDH particles in an aqueous chloride solution contributes to unveiling the initial stages of the process.

Recently, our research group developed an MD framework [6] based on a straightforward procedure and an open-source simulation package capable of exploring the structure and dynamics of periodic models of LDHs intercalated with different inorganic anions (nitrate, chloride and carbonate) for long simulation periods. Despite all atomic positions being allowed to move freely during the simulations, the integrity of the whole LDH structure was kept intact, and the structural parameters of the crystalline structure (namely, the distance between cationic layers and the distance between metallic atoms in the cationic layer) were found to be in good agreement with experimental X-ray diffraction (XRD) results. In addition, taking as an example the application of LDHs as nanostructured reservoirs applied in corrosion protection, the consistency of the MD framework and the model was supported by the complete immersion of LDH particles, with the nitrate anion intercalated (LDH-NO_3_) in a sodium chloride water solution, which was demonstrated experimentally to act as a nanotrap for aggressive anions when LDH-NO_3_ nanoadditives are included in coatings [16]. The structural integrity of the LDH-NO_3_ structure was also maintained throughout the MD simulation, even though the LDH particles were free to move alongside the solution, allowing a natural anion exchange between the LDH and the solution, as well as dehydration/hydration of the basal space [6]. That work served as a proof of concept, despite the observed anion exchange being limited. In this work, the LDH particle model and surrounding conditions were optimized so that more extensive anion exchange could be observed. The MD framework developed in those earlier works from our research group [6,7] has already impacted other works in literature dealing with LDHs, such as the simulation of the removal of hazardous materials [21], the examination of the degree of undulation of the cationic layers of exfoliated LDHs [22], the optimization of the steps involved in the MD procedure to evaluate the structure of LDHs as drug nanocarriers [23], and the partial charge parameterization of the MD force field to evaluate the hydration states of interlayer contents of different LDH materials [24].

Afterward, the MD framework described above was implemented to unveil the interlayer structure of an LDH material intercalated with an efficient corrosion inhibitor [25], benzo[d]thiazole-2-thiol (a.k.a. 2-mercaptobenzothiazole, MBT) [7]. In that work, it was examined the position of MBT anions in the interlayer, together with their conformational and tautomeric equilibrium [26], as well as the degree of solvation within the interlayers [7].

In this work, in order to understand the anion exchange process involving LDHs, MD simulations were performed using LDH-NO_3_ and LDH-MBT model particles in an aqueous sodium chloride solution. A detailed overview of the initial stages of the anion exchange mechanism was provided while observing the changes in the LDH structure during the process.

## 2. Computational Methodology

Classical MD simulations were carried out with the Gromacs 5.1.4 open software [27], using a leapfrog algorithm [28] to integrate the equations of motion and time steps of 0.5 or 1 fs for the equilibration and production stages, respectively. The non-bonded interactions considered Lennard-Jones (LJ) and electrostatic interactions calculated up to a cut-off of 1.4 nm. A potential force-switch modifier function forcing the energy to decay smoothly to zero between 0.8 to 1.4 nm was used for the LJ term, while the long-range electrostatic interactions were calculated through a combination of Particle Mesh Ewald (PME) [29] and a Coulomb potential-shift function. The energy contributions to the potential energy function from the bonded interactions considered bond stretching, angle bending and dihedral torsion terms, with all bond lengths constrained by the LINCS algorithm [30].

The temperature was fixed at 298 K, with the velocity-rescaling and Nose-Hoover thermostats for equilibration and production runs, respectively [31,32]. The pressure coupling was considered as semi isotropic in XY and Z directions, and the pressure was fixed at 1 bar using the Berendsen pressure-coupling method [33] for the equilibration steps and the Parrinello−Rahman barostat [34] for the production runs.

The LJ parameters were taken from our previous work [6], which employed a modified version of the CLAYFF force field [35] for Zn atoms in the LDH structure and a new set of atomic point charges, calculated after density functional theory calculations employing periodic models of the LDH structure. The SPC/E potential [36] was selected for water because of its favorable ratio between computational tractability and accuracy of calculated properties when compared with experimental results for density, O-O and O-H pair-distribution functions and self-diffusion coefficients [37]. The force field parameters for negatively charged MBT anion were adapted from those in the Automated Topology Builder (ATB) repository [38], based on the GROMOS force field [39], with atomic partial charges obtained from separate B3LYP/6-31G* calculations and the CHelpG approach.

**Figure 1:**
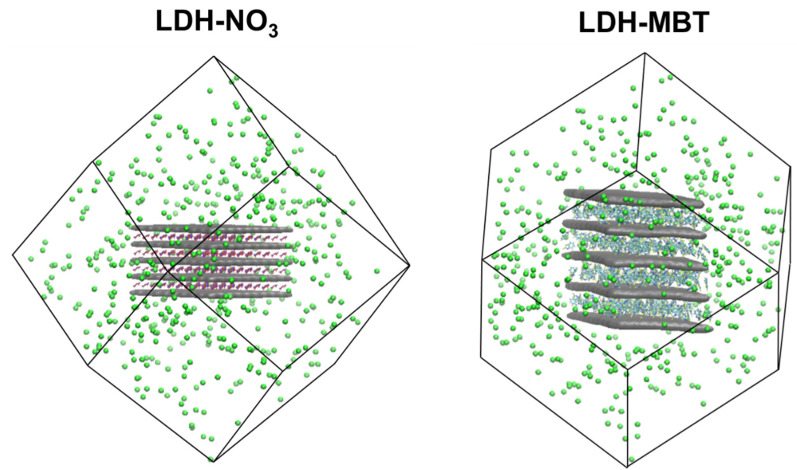
Initial models of LDH-NO_3_ (**left**) and LDH-MBT (**right**) were used to perform the release kinetics MD simulations. The cationic layer isosurface is presented in grey, chloride atoms in green, nitrogen in blue, oxygen in red, carbon in turquoise, sulphur in yellow, and hydrogen in white.

The initial structures of LDH-NO_3_ and LDH-MBT (Figure 1) were taken from previous studies, in which they were validated using XRD and thermogravimetric analysis (TGA) results [6,7]. These are composed of 500 units of [Zn(2)Al(OH)_6_]^+^ distributed uniformly forming 5 cationic layers, with the NO_3_^−^ and MBT^−^ (400 negative charges) present only in the interlayers, in order to devote this study only to interlayer anion exchange effects. Therefore, the outer 100 positive charges of the cationic layers were compensated by 100 chloride anions on the external surface of the LDH. The LDH models were centered in a dodecahedron box and surrounded by approximately one nanometer of empty space for solvation. The same number of interlayer species (NO_3_^−^ or MBT^−^) was inserted in terms of chloride anions for substitution and sodium cations to compensate for the surrounding chloride charges. Furthermore, the interlayer was filled with water molecules using the tools provided in the Gromacs package (insert-molecules and solvate). The simulation boxes contain the same number of water molecules for keeping the concentration constant, with the number of each species in each simulation being provided in Table 1. The same minimization and equilibration protocol was followed for Zn(2)Al-NO_3_ and Zn(2)Al-MBT LDHs. Energy minimization was carried out in two steps: firstly, the whole LDH was constrained, which was followed by another minimization step where only the metal atoms were frozen. After the energy minimization, two NVT simulations were done to equilibrate the initial temperature, one fixing the positions of the metal atoms (1 ns) followed by another with all atoms unconstrained (2.5 ns). Then, 10 ns of NpT simulation were performed to equilibrate the volume, which was followed by 100 ns of NpT simulation to produce the trajectory with all atomic positions unconstrained used in the subsequent analyses.

The analysis of the results was performed with a python code developed in-house (https://github.com/gnovell/LDH_MBT_codes/blob/master/XRD_parallel.py, accessed on 13 September 2022). The simulation of the XRD pattern for each simulated system was made using Debye’s formula [40], where the thermal vibration of atoms is considered upon the introduction of a damping exponent factor (Debye-Waller factor), and the angular dependency of geometrical and polarization factors are expressed as suggested by Iwasa et al. [41], and used in one of our previous studies [7].

The exchange of the ions from the LDH galleries to the surrounding volume and vice-versa was followed with another in-house code (https://github.com/gnovell/Counter_LDH_Cluster/blob/main/CounterAtoms.py, accessed on 1 October 2022) that counts the ions of the interlayer, such as nitrates. The code uses the DBSCAN algorithm of the scikit-learn module [42] to locate the “cluster” of Al^3+^ and Zn^2+^ hydroxide layers for each frame of the MD simulation and outturns the volume of the cluster and counts the species in the LDH galleries, i.e., water and anions. This counting makes use of the Qhull library of SciPy for determining the convex hull and then locates the positions inside the hull [43,44].

## 3. Results and Discussion

In previous works, Zn(2)Al LDH structures with the nitrate and MBT^−^ anions intercalated were successfully simulated and found in agreement with experimental XRD and TGA results while unveiling conformational, solvation and other inner structure details [6,7]. In this work, MD simulations were used to understand the anion exchange processes of both LDH materials at an atomistic level, using Zn(2)Al-NO_3_ and Zn(2)Al-MBT models inserted into water boxes containing a realistic number of Na^+^/Cl^−^ species. In both cases, a small model of an LDH particle was immersed into a periodic simulation box of salted water (please refer to Table 1 for details about the number of species), corresponding to a NaCl concentration of approximately 322 mM. This concentration is between 5 mM and 500 mM, which are concentrations commonly used in experimental tests to simulate a corrosive environment [16].

**Figure 2:**
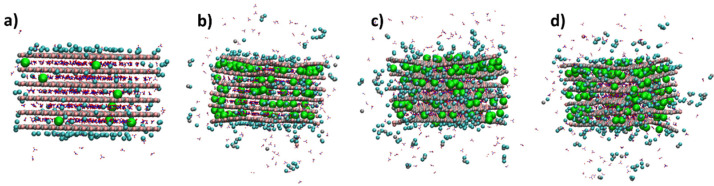
Snapshots of the different stages of the MD simulation for LDH-NO_3_. After the NVT equilibration ((**a**), 0 ns), NpT equilibration ((**b**), 10 ns), and the two NpT production runs of 50 ns ((**c**), 60 ns and (**d**), 110 ns). The chemical elements’ color code is ochre for Al, silver for Zn, red for O, and white for H. Cl inside the LDH is in green, while cyan was used for external Cl (surface Cl) at less than 5 Å of Al and Zn. The nitrates are represented by dots and sticks with blue for N and red for O. Magnified versions of the panels presented in this Figure are provided in the SI (Figure S1).

**Figure 3:**
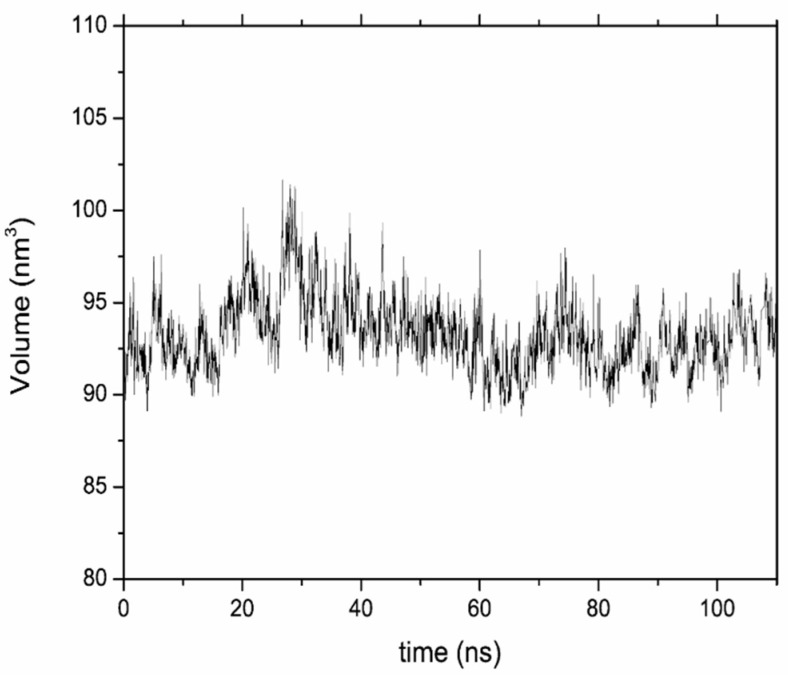

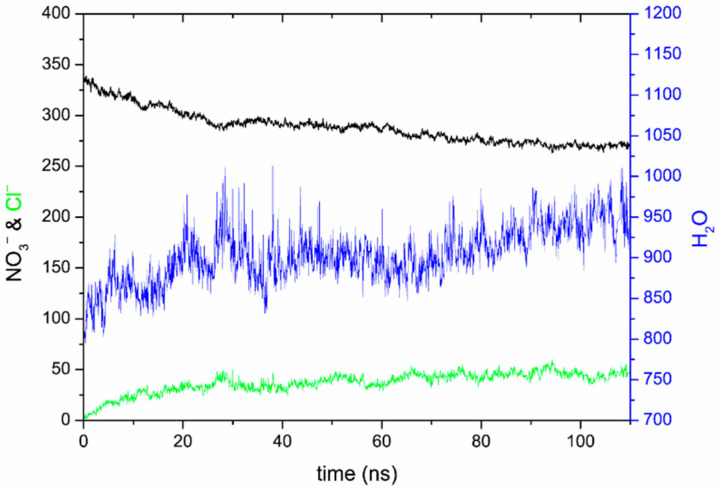
Volume (in nm^3^) of the LDH-NO_3_ model particle calculated with resort to the positions of the Al^3+^ and Zn^2+^ ions in the cationic layers in each frame of the MD simulation (**top**). The number of nitrate anions (black), chloride anions (green), and water molecules (blue) within the LDH-NO_3_ interlayers (**bottom**).

Selected views along the MD simulation performed for LDH-NO_3_ are shown in Figure 2. First, an NVT equilibration was performed. After this NVT equilibration, the time count was initiated, and 10 ns of NpT equilibration were performed, followed by 100 ns of NpT production. Views at the end of the NpT equilibration (Figure 2b) and at the middle (Figure 2c) and end (Figure 2d) of the NpT production stage show the progressive exchange of nitrate by chloride anions. During the initial NVT equilibration, it was already possible to see nitrate anions at the edges of the LDH model particle exiting the surrounding solution and a couple of chloride anions at the edge of the LDH particles (Figure 2a). After the start of the NpT equilibration (*t* = 0 in Figure 3), the chlorides entered the interlayer and diffused towards the inner parts of the LDH galleries (Figure 2b), which was followed by visible undulations of the LDH layers accompanying the process of the ionic interchange (Figure 2c) until the end of the simulation Figure 2d). Although the average volume only increased slightly between the beginning and the end of the simulation, the undulation resulted in an increase of volume variation from step to step, along the simulation depicted in the left panel of Figure 3. Accounts of undulation effects for LDHs were previously reported in the literature [6,45,46]. The slight increase in volume and the high volume variation from step to step were accompanied by a small increase in the number of water molecules counted inside the galleries, as well as a high variation of water molecules from step to step. At the end of the simulation period considered in this work, the number of nitrate anions counted within the LDH interlayers decreased by ~20%, accompanied by a similar increase in the number of chloride anions inside the LDH (Figure 3, bottom), which had to be expected for counterbalancing the positive charge of the metal hydroxide layers (a.k.a. cationic layers). Besides the undulation of the cationic layers, it is also possible to notice some opening of the cationic layers in the extremities of the LDH-NO_3_ particles during the release of nitrate in exchange by chloride (Figure 2d). This can be caused by the hydration of the outer parts of the particles during the process, as evidenced by the increase of the water molecules in the interlayer (Figure 3), which might promote a residual but detectable dissolution of the cationic layers, as evidenced by high-performance liquid chromatography measurements [4] and previous MD studies [6].

**Figure 4:**
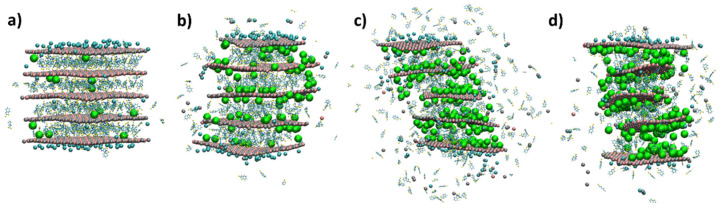
Snapshots of different stages of the MD simulation for LDH-MBT. After the NVT equilibration ((**a**), 0 ns), NpT equilibration ((**b**), 5 ns), and the production runs of 50 ns ((**c**), 60 ns and (**d**), 110 ns). The chemical elements’ color code is ochre for Al, silver for Zn, red for O, and white for H. Cl inside the LDH are in green, while cyan was used for external Cl (surface Cl) at less than 5 Å of Al and Zn. The MBT^−^ anions intercalated in the LDH galleries are represented with a ball-and-stick model, using blue for N, yellow for S, cyan for C, and white for H. Magnified versions of the panels presented in this Figure are provided in the SI (Figure S2).

**Figure 5:**
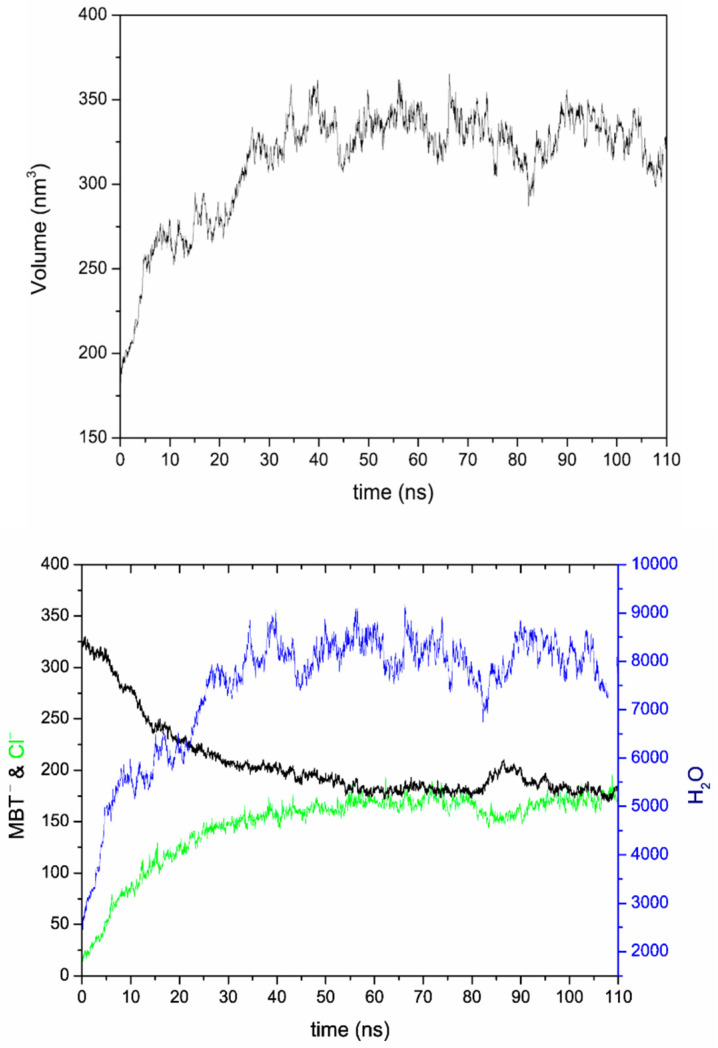
Volume (in nm^3^) described by the metal atoms (Al and Zn) position corresponding to the cationic layers of the LDH-MBT model particle in each frame of the MD simulation (**top**). The color of the molecular species inside the LDH-MBT interlayers is black for 2-mercaptobenzotiazole in the anionic form (MBT^−^), green for chloride anions, and blue for water molecules (**bottom**).

The MD simulations of Zn(2)Al-MBT performed in this work were able to elucidate what happened at the atomic level when the MBT^−^ corrosion inhibitor was released from the interlayers, triggered by the presence of aggressive chloride anions, which were subsequently intercalated into the LDH model particle. This phenomenon constitutes the first part of the active corrosion protection mechanism involving LDHs [18]. The anion exchange process is similar to what was described above for LDH-NO_3_. However, a more pronounced delamination process can be observed in Figure 4, where the interlayer heights significantly increase with simulation time, accompanied by the rotation of the LDH cationic layers while maintaining their structural integrity. The analysis of the volume described by all LDH layers along the simulations within the NpT ensemble allows us to quantify the delamination process, resulting in an increase in volume during the anion exchange process, as can be seen in Figure 5 (top). This volume increase for LDH-MBT during anion exchange is also accompanied by an increase of water molecules entering the interlayer. More specifically, ~8200 water molecules enter the interlayer during the anion exchange process for LDH-MBT, whereas for LDH-NO_3_, only ~800 enter the interlayer.

The LDH-MBT delamination requires the breakage of the hydrogen bonding between the cationic layers and the interlayer contents. To achieve the exfoliation of the LDH cationic layers in the form of nanosheets, traditional approaches usually involve the pre-intercalation of larger species to induce the swelling between cationic layers [47,48]. Therefore, the fact that LDH-MBT^−^ shows a larger degree of delamination during the anion exchange process than nitrate is in agreement with early experimental methodologies employed to exfoliate LDHs. Moreover, in a previous periodic model density functional theory study from our research group, it was also shown that larger interlayer distances could be associated with more favourable delamination energies [5].

The presence of delamination during anion exchange also finds support from a previous study, in which anion exchange processes in LDHs were investigated by in situ X-ray diffraction [49]. In that study, it was demonstrated that the exchange reactions lead to a decrease in the average crystallite size of LDHs, which, according to the authors, occurs due to the fast anion exchange in the initial stage, as was also verified in this MD study (Figure 5). The crystallite size was measured according to the full with at half maximum of the (006) diffraction peak, which is associated with the size of the crystallites perpendicular to the cationic layers.

In order to analyse the anion exchange simulation beyond 110 ns of NpT for LDH-MBT, the MD simulation was prolonged an additional 50 ns for this system with the results presented in the SI (Figures S3–S5). It was verified that the number of MBT^−^ anions, chlorides and water molecules in the interlayer appear to be stabilizing after an initial fast release, with further anion exchange progressing more slowly from thereon. Indeed, extensive anion exchange of LDH-MBT materials usually takes around 30 min (although experiments are run for at least a few hours or even a couple of days to ensure complete substitution) [50]. On the one hand, only the initial stage of the substitution within very short MD timeframes (160 ns) was analysed, but, on the other hand, this timeframe is proportional to the nanosized model of the LDH particle considered in this work. Therefore, in future works, other factors can be considered, such as temperature and a larger simulation box, to reduce the effect of changes in the concentration of the anions during the anion exchange process while increasing the number of surrounding anions available for substitution. Moreover, this initial atomistic MD study can guide the future development of a coarse-grain model, which might be more computational efficient for examining larger systems and longer time frames [51].

When comparing the number of chloride anions entering the interlayer in Figure 5 (bottom) for MBT with Figure 3 (bottom) for nitrate, it can be observed that the anion exchange process for a larger molecule such as MBT^−^, with a more pronounced delamination mechanism for the same period of time, seems to be faster than for nitrate (at least, for the nanoscale in terms of size and time of the models used in this study). This observation is supported by an in situ kinetic study of the anion exchange in LDHs using synchrotron XRD, where it was verified that a larger initial interlayer gallery in comparison with the entering anion corresponds to a faster substitution [52], with other studies also supporting this observation [53]. Moreover, radial distribution function (RDF) analysis can be observed in SI (Tables S1 and S2, and Figure S6).

## 4. Conclusions

The MD computational model of the initial stages of the anion exchange process in LDHs allowed us to identify delamination as a key factor in the mechanism. MD simulations were employed in this work to understand the anion exchange component of the active corrosion protection ability of LDHs, comparing the release of nitrate with a typical corrosion inhibitor, 2-mercaptobenthiazole, while aggressive chloride anions were retrieved from solution to be intercalated in the LDHs. It was found that the release of MBT^−^ was accompanied by a more pronounced delamination effect, which seems to be dependent on the size of the initial anions present in the LDH galleries. The molecular dynamics simulations provided atomistic-level insights into the anion exchange process, which are very difficult to obtain from experimental techniques alone. Indeed, it was possible to model by MD simulations previous observations regarding undulation effects [6,45,46], stability of the cationic layers [4,6], reduction of crystallite size during anion exchange [49], the promotion of exfoliation by delamination in LDHs [5,47,48], and the relation between the initial interlayer gallery and the velocity of anion exchange [52,53].

## Figures and Tables

**Table 1:** Composition of the LDH systems studied in this work.

Model	Zn(2)Al-NO_3_	Zn(2)Al-MBT
LDH	500	500
NO_3_^−^	400	
MBT^−^		400
Water *	800/80,252	1800/79,252
Cl^-^	500	500
Na^+^	400	400

* Number of water molecules inside/outside the Zn(2)Al-LDH galleries.

## Data Availability

The data used in this study are available in the Supplementary Materials or from the corresponding authors upon reasonable request.

## Supplementary Materials

The following supporting information can be downloaded at: https://www.mdpi.com/article/10.3390/nano12224039/s1, Figure S1: Large scale version of the snapshots of the different stages of the MD simulation for LDH-NO_3_, Figure S2: Large scale version of the snapshots of the different stages of the MD simulation for LDH-MBT, Figure S3: Volume (in nm^3^) described by the position of the metallic atoms (Al and Zn) corresponding to the cationic layers of the LDH-MBT model particle for the extra 50 ns of MD simulation, Figure S4: Number of water molecules inside the LDH-MBT interlayers for the extra 50 ns of MD simulation, Figure S5: Number of chloride and MBT anions inside the LDH-MBT interlayers for the extra 50 ns of MD simulation, Figure S6: LDH figure representing the center in purple used as a reference for the RDF analysis, Table S1: Analysis for LDH-NO_3_ of the first RDF peak of different species using as a reference the center of the cationic layers, Table S2: Analysis for LDH-MBT of the first RDF peak of different species using as a reference the center of the cationic layers.
