# Supplementary material for: Molecular Dynamics Model to Explore the Initial Stages of Anion Exchange involving Layered Double Hydroxide Particles
Source: Nanomaterials (Basel). 2022 Nov 17;12(22):4039. doi: 10.3390/nano12224039 (PMC9695576; doi:10.3390/nano12224039)
Supplement: Supplementary file 1 [file nanomaterials-12-04039-s001.zip › nanomaterials-2000576-supplementary.pdf]

SUPPORTING INFORMATION

Molecular Dynamics Model to Explore the Initial Stages of Anion Exchange involving Layered Double Hydroxide Particles

Gerard Novell Leruth [1,2], Alena Kuznetsova [2,3], João Tedim [2], José R. B. Gomes [1,*] and Tiago L. P. Galvão [2,*]

[1] CICECO-Aveiro Institute of Materials, Department of Chemistry, University of Aveiro, Campus Universitário de Santiago, 3810-193 Aveiro, Portugal
[2] CICECO-Aveiro Institute of Materials, Department of Materials and Ceramic Engineering, University of Aveiro, Campus Universitário de Santiago, 3810-193 Aveiro, Portugal
[3] Smallmatek—Small Materials and Technologies Lda., Rua dos Canhas, 3810-075 Aveiro, Portugal
* Correspondence: jrgomes@ua.pt (J.R.B.G.); tlpgalvao@ua.pt (T.L.P.G.)

The supporting information includes large scale snapshots of the MD simulations, results for the extra 50 ns simulations performed for LDH-MBT, and radial distribution function (RDF) information.

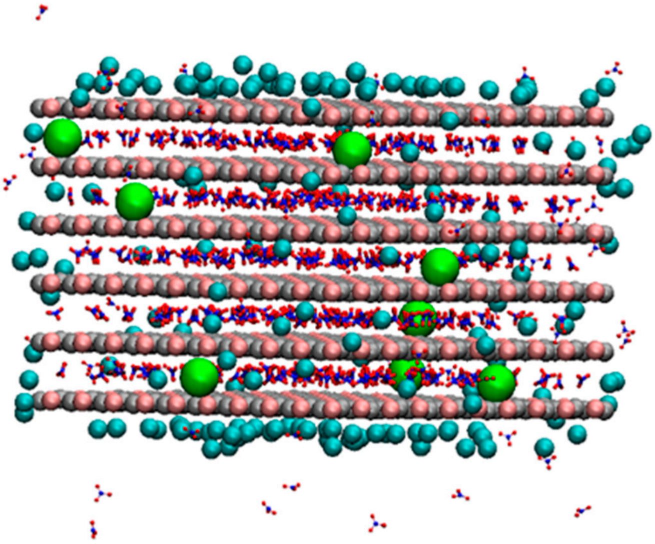

0 ns

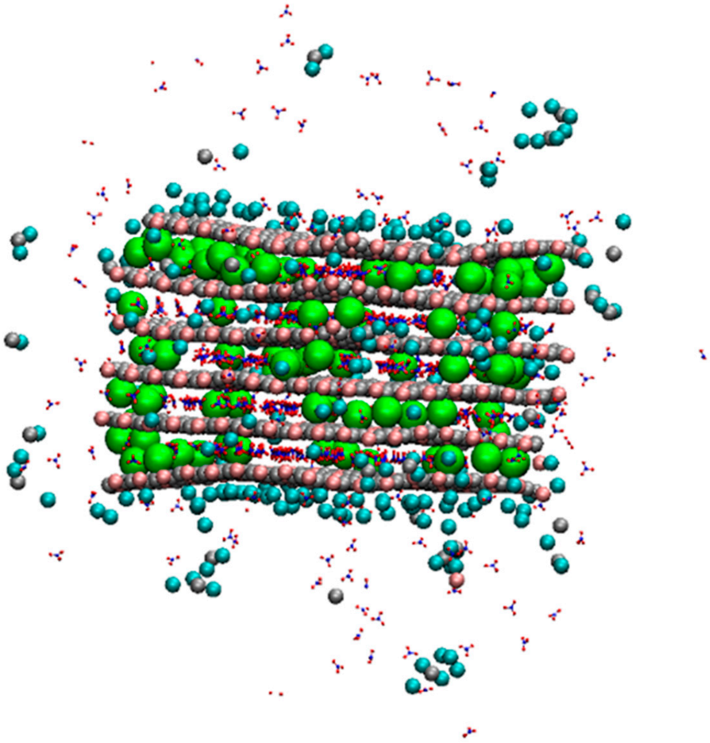

10 ns

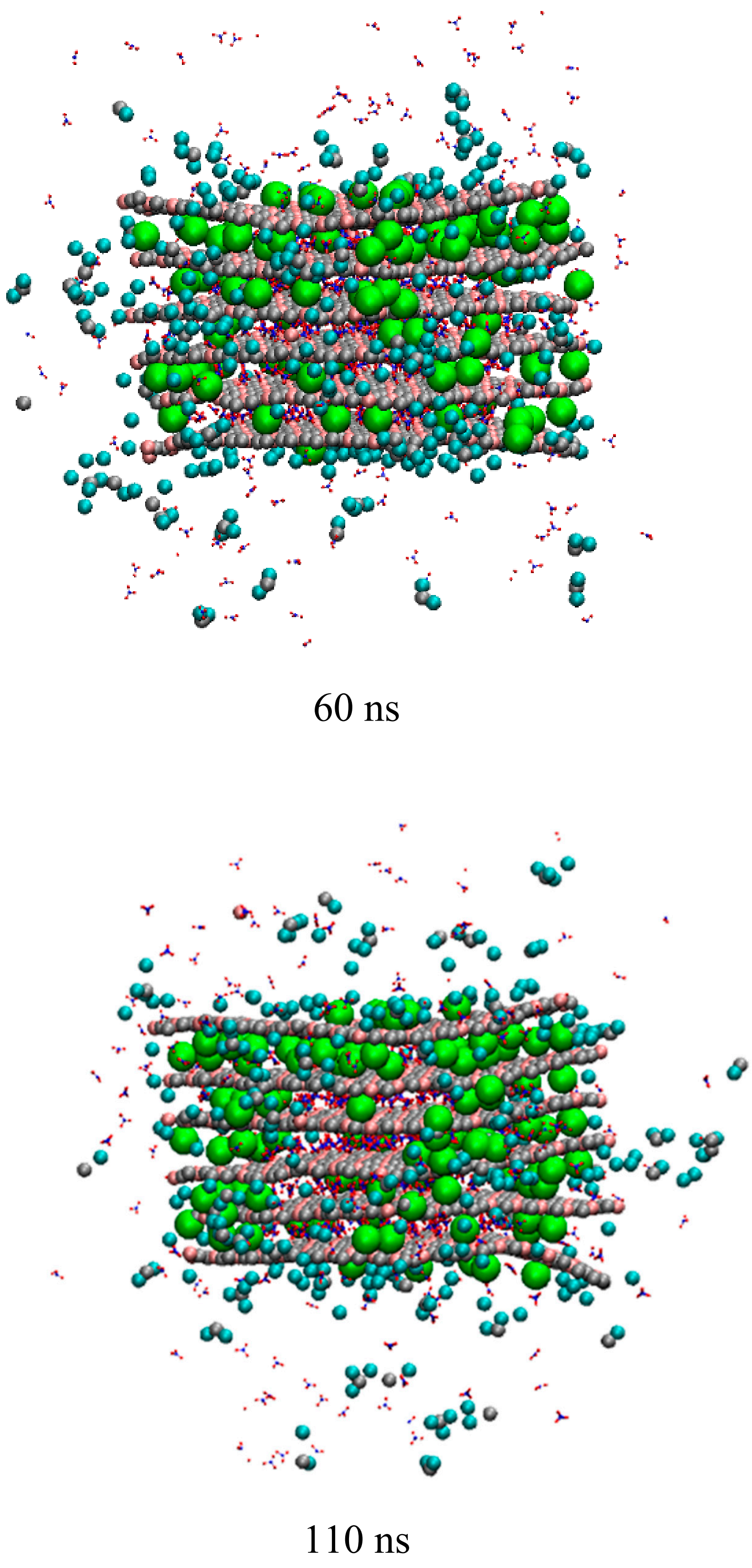

Figure S1. Large scale version of the snapshots of the different stages of the MD simulation for LDH-NO_3.

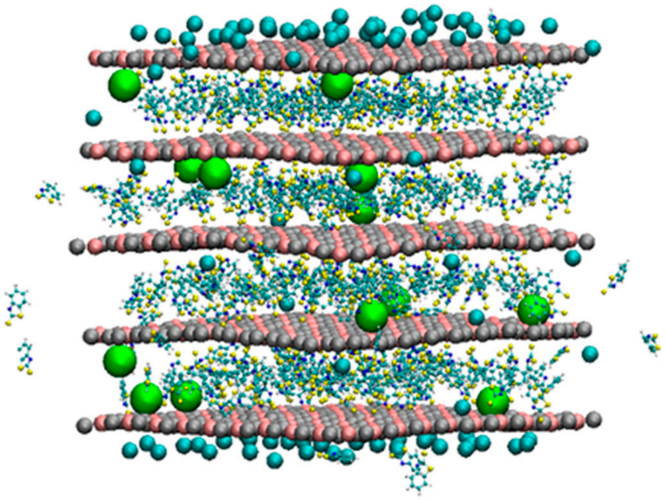

0 ns

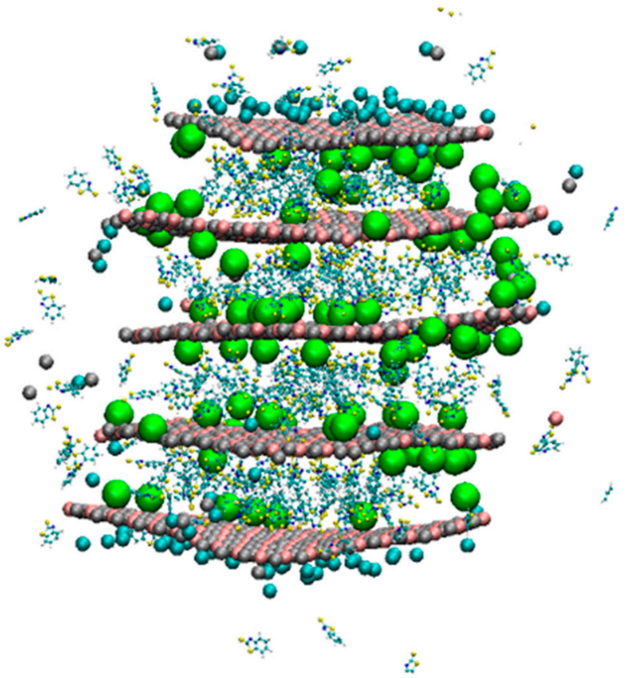

10 ns

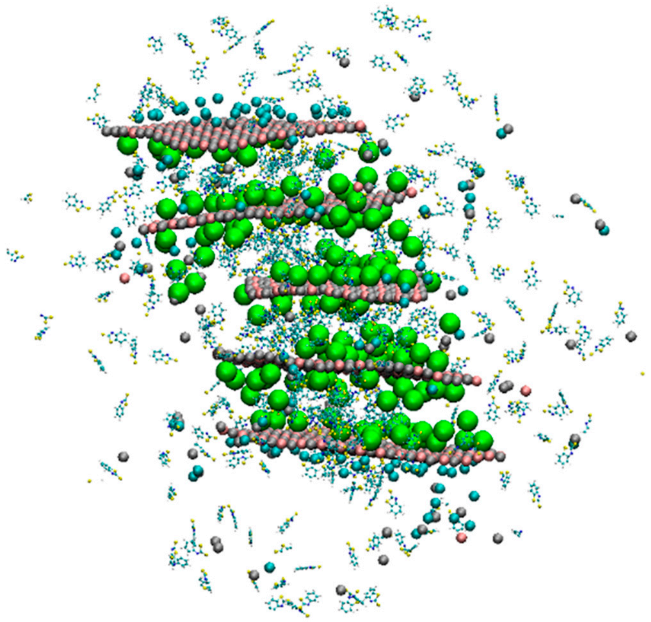

60 ns

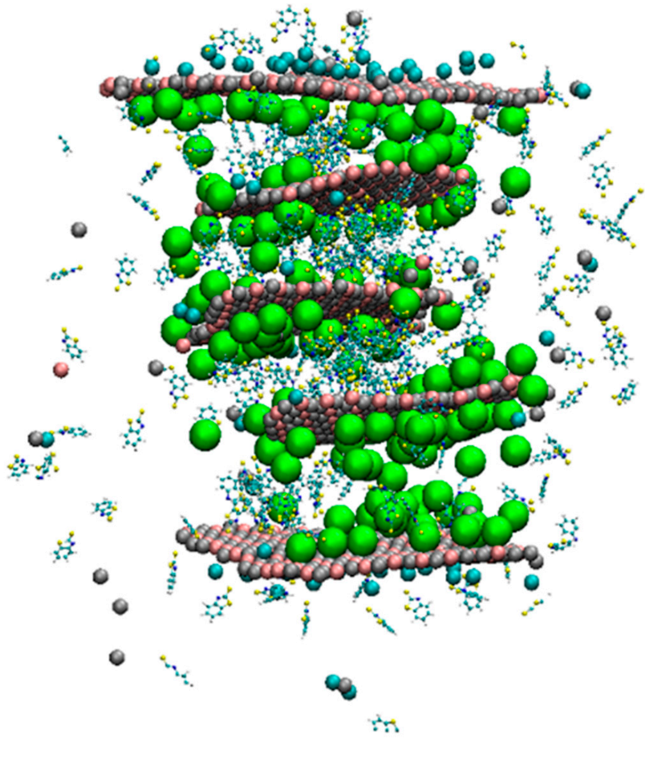

110 ns

Figure S2. Large scale version of the snapshots of the different stages of the MD simulation for LDH-MBT.

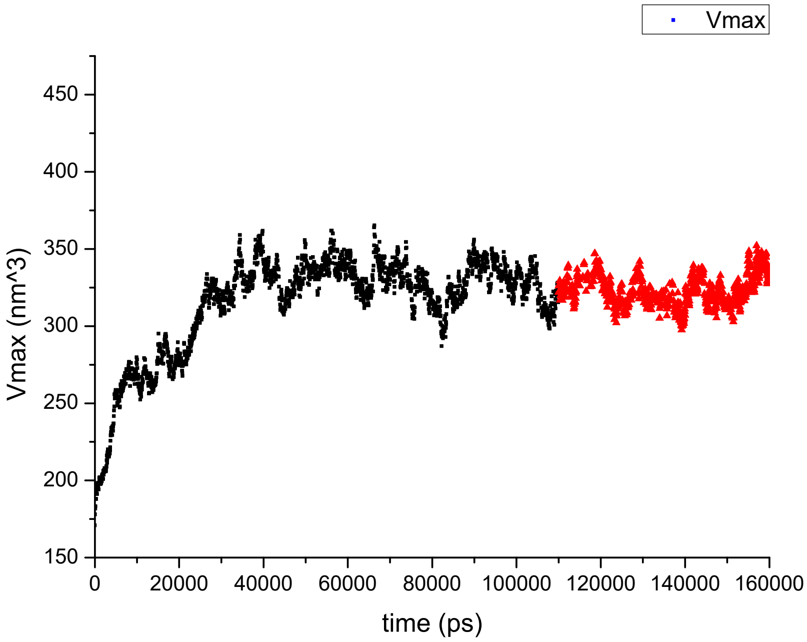

Figure S3. Volume (in nm^3) described by the position of the metallic atoms (Al and Zn) corresponding to the cationic layers of the LDH-MBT model particle for the extra 50 ns of MD simulation.

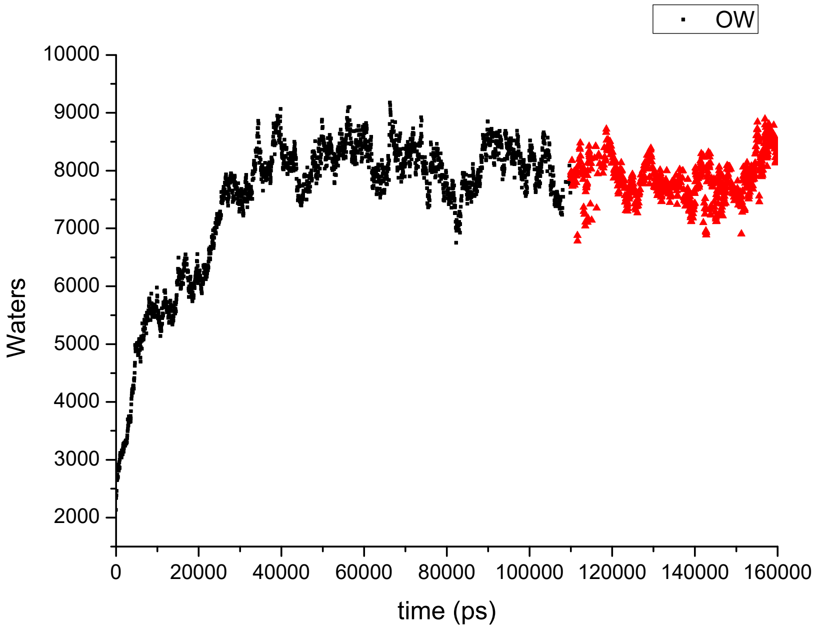

Figure S4. Number of water molecules inside the LDH-MBT interlayers for the extra 50 ns of MD simulation.

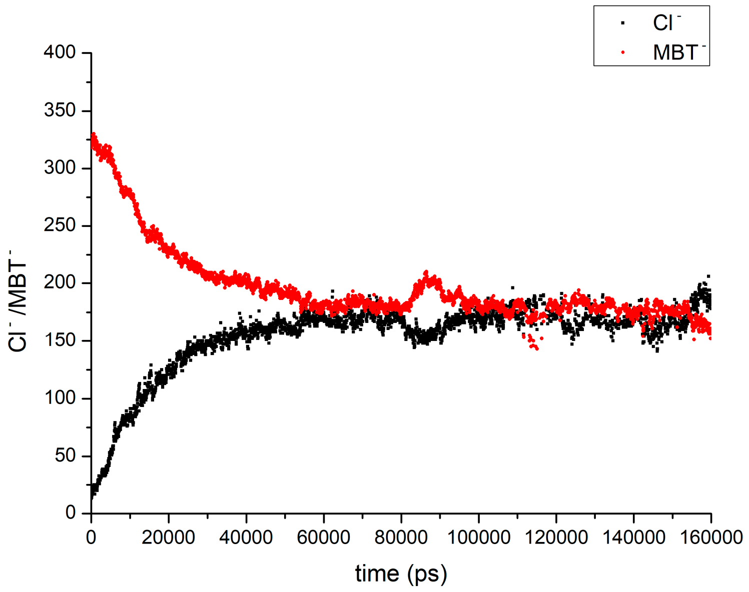

Figure S5. Number of chloride and MBT anions inside the LDH-MBT interlayers for the extra 50 ns of MD simulation.

Table S1. Analysis for LDH-NO_3 of the first RDF peak of different species using as a reference the center of the cationic layers.

	Metallic atoms		N from NO_3^-		Cl^-		O from H_2O	
time (ns)	r(nm)	g(r)	r(nm)	g(r)	r(nm)	g(r)	r(nm)	g(r)
0	0.306	961.487	0.434	609.665	2.178	26.884	2.096	0.483
10	0.308	304.045	0.506	442.046	2.15	70.497	2.088	0.434
60	0.304	314.288	0.498	452.951	1.446	48.899	0.368	12.2
110	0.31	288.765	0.458	530.873	1.55	42.272	0.442	8.996

Table S1 represents the analysis of the first peak of the RDF using as a reference the center of the cationic layers, as evidenced in Figure S6. The distance of the metallic atoms (Al and Zn) to the center is practically stable. It is possible to notice that the chloride anions enter the LDH, but the maximum density is still around 1.5 nm of the center. The water molecules also enter the LDH, but those that solvate the interlayer anions are much lower number than the water molecules that remain on the outside of the LDH, and for this reason its probability is low.

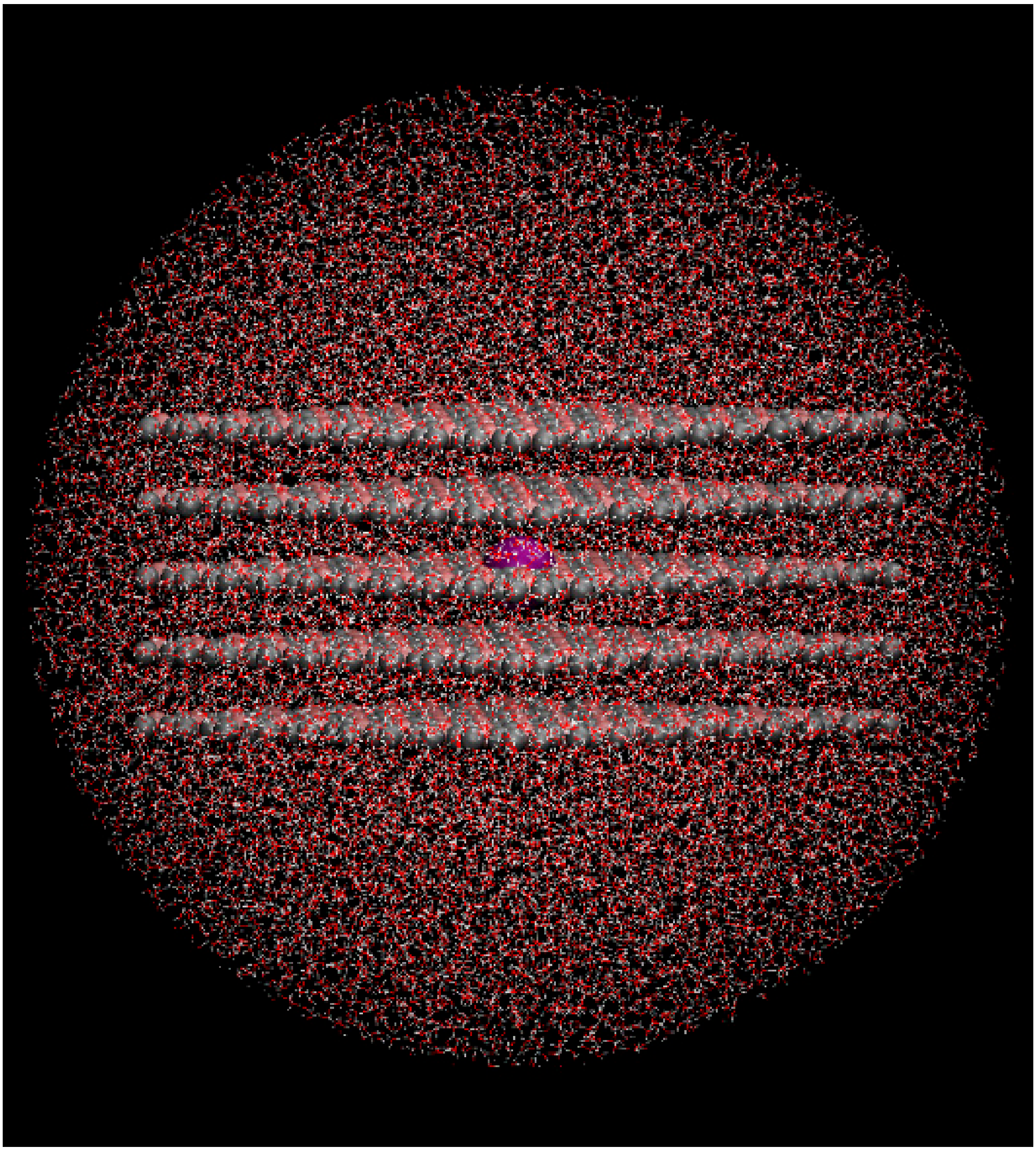

Figure S6. LDH figure representing the center in purple used as a reference for the RDF analysis.

Table S2. Analysis for LDH-MBT of the first RDF peak of different species using as a reference the center of the cationic layers.

	Metallic atoms		S from MBT^{--}		Cl^-		O from H_2O	
time (ns)	r(nm)	g(r)	r(nm)	g(r)	r(nm)	g(r)	r(nm)	g(r)
0	0.316	1404.951	0.608	722.418	1.926	107.034	0.386	19.236
10	0.302	751.216	0.586	828.077	0.709	583.516	0.358	16.939
60	0.304	693.771	0.574	632.657	0.868	228.602	0.372	6.797
110	0.312	1196.712	0.538	948.308	0.646	509.727	0.384	11.487

In the case of the LDH-MBT RDF analysis, it is possible to verify that the chlorides were introduced in the LDH with similar distances as MBT. The amount of water in LDH-MBT is higher than in the case of LDH-NO_3, and this is reflected in a lower distance of the highest probability to the center in the case of LDH-MBT.

All RDF files and retrieved data are available online:

Galvao, Tiago (2022), “Molecular Dynamics Model to Explore the Initial Stages of Anion-Exchange involving Layered Double Hydroxide Particles - RDF data”, Mendeley Data, V1, doi: 10.17632/pfvwhs7xzb.1
